# Supplementary material for: The Mexican magnetic resonance imaging dataset of patients with cocaine use disorder: SUDMEX CONN
Source: Sci Data. 2022 Mar 31;9:133. doi: 10.1038/s41597-022-01251-3 (PMC8971535; doi:10.1038/s41597-022-01251-3)
Supplement: Supplementary file 2 [file 41597_2022_1251_MOESM2_ESM.docx]

Angeles-Valdez *et al.* Supplementary material

**The Mexican magnetic resonance imaging dataset of patients with cocaine use disorder: SUDMEX CONN**

**Supplementary 2**

| **Supplementary 2. Eliminated participants**  **RID Elimination criterion**  37 No MRI study.  39 Diagnosis of Diabetes Mellitus (DM).  63 No cognitive/clinical data.  69 Diagnosis of Diabetes Mellitus (DM).  88 No cognitive/clinical data.  102 Diagnosis of Diabetes Mellitus (DM) and hypertension (HPN) and no  cognitive/clinical data.  119 Diagnosis of hypertension (HPN) and no cognitive/clinical data. |
| --- |
