# Supplementary material for: The Mexican magnetic resonance imaging dataset of patients with cocaine use disorder: SUDMEX CONN
Source: Sci Data. 2022 Mar 31;9:133. doi: 10.1038/s41597-022-01251-3 (PMC8971535; doi:10.1038/s41597-022-01251-3)
Supplement: Supplementary file 3 [file 41597_2022_1251_MOESM3_ESM.docx]

Angeles-Valdez *et al.* Supplementary material

**The Mexican magnetic resonance imaging dataset of patients with cocaine use disorder: SUDMEX CONN**

**Supplementary 3**

| **Supplementary 3. Study criteria.** |
| --- |
| **Inclusion**   - Minimum age of 18 years and maximum of 50 years old (CUD/HC). - Cocaine use for at least 1 year, with current average use of at least 3 times a week, with periods of continuous abstinence of less than one month during the last year (CUD). - Desire to participate and agree to the informed consent (CUD/HC). |
| **Exclusion**   - First-degree personal or family history of any clinically defined neurological disorder (HC). - Any electronic or metal implants or device (i.e., aneurysm clips, shunts, stimulators, cochlear implants, or electrodes) (CUD/HC). - Splinters of metal or metal projectiles to the head or body (CUD/HC). - Current use of any investigational drug or of any medicine with anti- or pro-convulsive action such as tricyclic antidepressants or neuroleptics, unless prescribed for craving symptoms (HC). - History of schizophrenia, bipolar disorder, mania, or hypomania (CUD/HC). - History of any heart condition currently under medical care (i.e., myocardial infarction, angina pectoris, congestive heart failure, etc.) (CUD/HC). - Women with reproductive potential not using an acceptable form of contraception, as well as pregnant or lactating women (CUD/HC). - Current dependence (by DSM-IV criteria) on substances other than cocaine and / or nicotine (cocaine use disorder) (CUD/HC). - Claustrophobia (CUD/HC). |
| **Elimination**   - Expressed desire to stop participating (CUD/HC). - Those who presented abnormal radiological findings warranting clinical attention outside the study to ensure the health of the participant (CUD/HC). - The appearance of psychotic symptoms related to addictive disorder (CUD). |
